# Supplementary material for: Deciphering the change in root system architectural traits under limiting and non-limiting phosphorus in Indian bread wheat germplasm
Source: PLoS One. 2021 Oct 1;16(10):e0255840. doi: 10.1371/journal.pone.0255840 (PMC8486105; doi:10.1371/journal.pone.0255840)
Supplement: S1 Table — (PDF) [file pone.0255840.s001.pdf]

Supplementary Table 1. List of 182 wheat genotypes used in the study.

Genotype	Pedigree
BW1	CSW2/HD2932+Yr15
BW2	HD2967/HD2887//HD2946/HD2733
BW3	HD3086/HD2887//HD3117/HD2967
BW4	HD2953/HS365
BW5	SAWYT-326/HD2967
BW6	CSW2/HD2932+Yr15
BW7	HD2789/HD2891//HD2932
BW8	HD2733/HD2887//HD2946/HD2932
BW9	HD2967/HD3117//HD3171/HD2733
BW10	HD2967/HD2887//HD3117/HD2733
BW11	HD3086/HD2329
BW12	VL849/NBP-39//HD2967
BW13	IM-15/HD2967
BW14	HD3117/HDCSW18
BW15	46thIBWSN-1236
BW16	ICSISA-SBMNP-4-158
BW17	ICSISA-SBMNP-2-152
BW18	CL1705/HD2687
BW19	SAWSN-3194
BW20	CL1591/CL1435//HD2967
BW21	HD2877/DW433//WH542/3/HD2982/2967
BW22	CSW114
BW23	HD2874/HD2967//43IBWSN-1087
BW24	DL5/PBW343//HD2891
BW25	SAWYT-326/HD2967
BW26	CL1705/HD2687
BW27	IRSBWYT-19
BW28	CSW16/ HD2932+Yr15Yr15//HD3086
BW29	CSW18/ HD2932+Yr15
BW30	HD3117/HD3171
BW31	HD2967/CP196
BW32	CSW2/Yr15
BW33	CL264/CL1633//CNo-601
BW34	CL204/CL1453//CNo-611
BW35	31-ESWYT-138/CSW30
BW36	CL1633/CNo-601
BW37	43IBWSN-1175
BW38	23rdHRWSN-2003
BW39	SAWYT-326/HD2967
BW40	CSW18/CSW1
BW41	CL1705/HD2967
BW42	HD3226/HD2329

BW43	HD3086/2784
BW44	HD2953/HS365
BW45	24thHRWSN-2112
BW46	VL616(2) Inqualab/Kundan
BW47	C306/HDCSW18
BW48	CL264/CL1633//CNo-601
BW49	43IBWSN-1175
BW50	31ESWYT-138/CSW30
BW51	CL1705/HD2687
BW52	CL2596/K9451//CL882/HD2009
BW53	31-ESWYT-135/3/HD2329/WR544//PBW343/NW3041
BW54	31ESWYT-135/HD2329
BW55	31ESWYT-135/CSW23
BW56	EBWYT-61
BW57	31stESWYT139/3/PBW343/PH 137//MC-11
BW58	SAWYT-326/HD2967
BW59	HD3086/HD2932//HD2329
BW60	VL332/Wei-132//VL829/3/VmI-39-II
BW61	HD3226/HD2967
BW62	CL1734/HD2643//HDK10/PBW502
BW63	HD3059/HD3054
BW64	C306//HI154/HD2967
BW65	31ESWYT-135/3/HD2329/WR544//PBW343/NW3041
BW66	HD3226/HD3086//HDCSW18
BW67	31-ESWYT-132/CSW17
BW68	31-ESWYT-132/CSW23
BW69	CSW2/ HD2932+Yr15
BW70	HD2967/HD2887//HD2946/HD2733
BW71	CSW18/CSW1
BW72	HD3226/HD2784//HD2946/HD2733
BW73	IBWSN//HD30/HD2824
BW74	18HRWYT214/18 HRWYT-229
BW75	31-ESWYT-147/3/HW5028//HD2432/DW2009
BW76	43IBWSN-1175
BW77	HD3189
BW78	C-47th IBWSN-405
BW79	CSW24/CSW26
BW80	HD2967//HD2887//HD2946/HD2733
BW81	CSW30/CSW34
BW82	22ndHRWSN-2112
BW83	HD2967//HD2887/HD2946//HD2733
BW84	47thIBWSN-24
BW85	HDCSW18/PBW677
BW86	HD2953/HS365

BW87	IM-15/HD2967
BW88	HD2967/RSP566
BW89	P50/K-35
BW90	IM-15/HD2967
BW91	ICSISA-SBMNP-4-158
BW92	CSW3/HD2932+Yr10
BW93	43IBWSN-1175
BW94	HD3117/HDCSW18//HD2932
BW95	WH542/UP2425//WH542/UP2425
BW96	HD3115/PBW550
BW97	31ESWYT-138/CSW23
BW98	CSW35/DBW17
BW99	HD3226/KALYANSONA
BW100	HD3086/NP5
BW101	IM-15/HD2967
BW102	CSW3/HD2932+ HD2932+Yr10
BW103	CSW18/CSW1
BW104	HD3117/HD3059
BW105	HD2789/HD2891//HD2932
BW106	CSW3/HD2932+Yr10
BW107	HD3086/HD2329
BW108	18HRWYT214/18 HRWYT-229
BW109	HD2967/HD2887//HD2946/HD3226
BW110	HD2877/DW343//WH542/3/HD2982/HD2967
BW111	HDCSW18/CSW1
BW112	HD2967//HDCSW16/HD3054
BW113	HDCSW16/CSW1
BW114	HD2784/HD3059
BW115	CSW18/CSW5
BW116	CSW3/HD2932+Yr10
BW117	HD3054/HD2967
BW118	HD3226/HDCSW16
BW119	HD CSW18/HD2329
BW120	HD3086//HD3059
BW121	IM-15/HD2967
BW122	CL1734/HD2643//HDK10/PBW502
BW123	HD2967/HDCSW16
BW124	HD2967/HD2887//HD2946/HD2733
BW125	31stESWYT-135/3/HD2329/WR544//PBW343/NW3041
BW126	CSW01/DBW17
BW127	CSW90
BW128	HD3115/PBW550
BW129	CSW18/HD2967
BW130	PBW504/VL849//43rdIBWSN-1164

BW131	CSW25/CSW18//HD2967
BW132	31ESWYT-138/3/PBW343//PH137/MC-11
BW133	UP2425/Blend-b-I-II
BW134	18HRWYT214/18 HRWYT-229
BW135	31ESWYT-135/CSW23
BW136	CSW3/HD2932+Yr10
BW137	HDCSW18/CSW1
BW138	CSW90/HD3086
BW139	HD2967/HD2887//HD2946/HD2733
BW140	CSW88
BW141	IM-15/HD2967
BW142	HD2329/HD2285//43IBWSN-1182
BW143	CSW2/ HD2932+Yr15
BW144	SAWYT-320/HD2967
BW145	SAWYT-326/HD3086
BW146	SAWYT-336/HD2967
BW147	IM-15/HD2967
BW148	HD3086//HD2786/HD3054
BW149	31 ESWYT-138/CSW23
BW150	HD2967/CP196
BW151	HD2967/HD2887//HD2946/HD2733
BW152	HDCSW18/HD3086
BW153	HD3226//HI154/C306
BW154	SAWYT-326/HD2967
BW155	HD2967/HD2887//HD2946/HD2733
BW156	HD3054/HD2887//CSW16/HD2733
BW157	HD2967/HD3117
BW158	HD2967/HD2887//HD2946/HD2733
BW159	31stESWYT135/CSW23/HDCSW18
BW160	31ESWYT-138/CSW30
BW161	IBWSN-85//HD2329/DW1293
BW162	CSW2/ HD2932+Yr15
BW163	HD2329/HDK10//CBW38/WR544
BW164	31ESWYT-132/CSW-17
BW165	HD2967/CP196
BW166	IM-15/HD2967
BW167	HD2967/HD2887//HD2946//HD2733
BW168	HD2874/HD2967//43rdIBWSN-1087
BW169	CSW3/HD2932+Yr10
BW170	CL1591/CL1435//HD2967
BW171	CSW2/ HD2932+Yr15
BW172	SAWYT-326/HD2967
BW173	HD2967/CP196
BW174	HD2967/HD2887//HD2946/HD2733

BW175	HD3054/HD2946//HD2733
BW176	HD2967/HDCSW18//NP4/HD2733
BW177	31ESWYT-135/CSW23
BW178	IM-36/HD2967
BW179	VL849/NBP-39//HD3056
BW180	HD3086//C306
BW181	CSW2/HDCSW18//HD 2784
BW182	C306/HD2329//HD2932/HD3059
