# Supplementary material for: Deciphering the change in root system architectural traits under limiting and non-limiting phosphorus in Indian bread wheat germplasm
Source: PLoS One. 2021 Oct 1;16(10):e0255840. doi: 10.1371/journal.pone.0255840 (PMC8486105; doi:10.1371/journal.pone.0255840)
Supplement: S2 Table — (PDF) [file pone.0255840.s002.pdf]

Supplementary Table 2. Comprehensive phosphorus response index value of 182 wheat advanced breeding lines used in the study.

s.no	Class	Range	Genotype
1	Highly responsive	>0.7	BW181, BW103, BW104, BW143, BW66.
2	Responsive	0.5-0.7	BW116, BW111, BW125, BW101, BW144, BW112, BW146, BW64, BW149, BW145, BW182, BW113, BW159, BW67, BW158, BW53, BW70.
3	Moderately responsive	0.3-0.5	BW150, BW176, BW175, BW22, BW120, BW61,BW13, BW115, BW18, BW59, BW174, BW128, BW129, BW153 BW177, BW121, BW117, BW99, BW63, BW28, BW119 BW14, BW148, BW107, BW142, BW147, BW102, BW154 BW169, BW110, BW105, BW83, BW152, BW180, BW69 BW109, BW108, BW156, BW160, BW65, BW72, BW71, BW114, BW155.
4	Low responsive	0.1-0.3	BW76, BW32, BW126, BW51, BW4, BW135, BW5, BW23, BW82, BW131, BW41, BW137, BW30, BW1, BW57, BW33, BW173, BW58, BW48, BW123, BW98, BW60, BW38, BW56, BW36, BW37, BW178, BW92, BW45, BW46, BW87, BW84, BW90, BW52, BW164, BW20, BW42, BW73, BW165, BW40, BW17, BW74, BW47, BW132, BW138, BW172, BW106, BW162, BW95, BW77, BW141, BW43, BW44, BW85, BW171, BW91, BW75, BW62, BW24, BW89, BW54, BW35, BW21, BW161, BW96, BW50, BW168, BW124, BW130, BW166, BW68, BW100, BW27,BW179, BW88, BW80, BW29, BW86, BW94, BW19, BW167,BW118,BW16,BW79, BW127, BW31, BW81, BW49, BW15, BW25, BW122, BW133, BW39, BW151, BW163, BW93, BW140, BW170, BW157, BW55.
5	Non responsive	<0.1	BW9, BW139, BW136, BW3, BW8, BW78, BW10, BW11, BW7, BW6, BW26, BW97, BW2, BW34, BW134, BW12.
