# Supplementary material for: Deciphering the change in root system architectural traits under limiting and non-limiting phosphorus in Indian bread wheat germplasm
Source: PLoS One. 2021 Oct 1;16(10):e0255840. doi: 10.1371/journal.pone.0255840 (PMC8486105; doi:10.1371/journal.pone.0255840)
Supplement: Attachment — Submitted filename: Comments_ Deciphering the change in root system architectural traits under limiting and non- limiting phosphorus in Indian bread wheat germplasm (1).docx [file pone.0255840.s003.docx]

**General comment:**

The work related to Root System Architecture (RSA) under Phosphorous limited conditions in wheat has not been studied comprehensively due to its difficulty in phenotyping. The manuscript reports identification of RSAtraits and genotypesinfluencingPuse efficiency under hydroponics conditions, which I feel a reliable strategy and could be used effectively for screening**.**These P efficient genotypes, as they are advanced and fixed lines, could serve as excellent donors in developing P efficient wheat genotypes. However, the manuscript need improvement especially in discussion part. **I would suggest moderate revision for this manuscript**. Specific comments are as follows.

**Introduction**

Introduction is well written. The importance of P in improving the productivity is well explained. However, authors should explain more about in RSA traits under P efficient and P limited conditions. If studies are limited in Wheat,other cereal crops may be explored like maize, rice etc. In rice, good number of papers are available on RSA traits influenced by P use efficiency (e.g. The role of root size versus root efficiency in phosphorus acquisition in rice. Mori et al 2013 Journal of Experimental Botany*,*2016, 67:1179–1189).

**Material and Methods**

- 182 advanced breeding lines comprising of diverse parentages is a good experimental material for this study.
- Statistical analysis is appropriate.
- Comprehensive phosphorus response index value is well calculated and is important identifying P responsive and non-responsive genotypes.
- Authors have not included released varieties in the experimental material Why?

**Result**

- The results are well presented.
- Subtitles should not be lengthy. The authors have written important findings in the subtitles which I feel quite good however, it will be confusing for the readers because of their lengthiness. Try to make it concise where it is possible or give name of the analysis as subtitle.
- In PCA, Figure 3B. the angle of eigen vectors between TRV, TRL, PRL, TRT, TRF and TSA is less than 90^o^, it indicates they are quite related to each other and we can take most important trait for the study leaving other less important traits. Which trait do you think is more important under limited P conditions and you advocate in the abstract for the readers?
- In title of figure 5 for better clarity, rather than writing RSA of contrasting genotypes, write better performing (BW 181 and BW 103) and poor performing (BW 9 and BE 139) genotypes grown under non-limiting (NLP) and limiting (LP) phosphorus conditions
- In P efficient genotypes, viz., BW181, BW103, and BW66 a common parent noticed is HDCSW 18. This is a very high yielding wheat variety developed by IARI, New Delhi for conservation agriculture conditions. It would be great if authors could write more about this variety in relation to its P or N use efficiency in results as well as in discussion.
- Similarly, in the parentage of P non-efficient wheat genotypes like BW 9, BW 139, BW 3, two mega wheat varieties HD 2967 and HD 2733 are involved. If authors would come out with a reason why recombinants of these best genotypes adapted to farmers field showed poor P efficiency? If authors would have involved released varieties (popular varieties like HDCSW 18, HD 2967, HD 3086, HD 2733 etc.) in this study, it would have been good information.

**Discussion**

- Discussion is quite lengthy and need to be revised.
- Results are repeated in the discussion which is not required.
- Authors can cite references from other cereal crops pertaining to RSA under different nutrient stresses.
- In the discussion, author may give statements on genetics of P use efficiency in studied genotypes based on their parentages e.g.
- Six genotypes shared common parentage (CSW2/ HD2932+Yr15); however, they differ for their P response index. BW143 is highly responsive, BW69 is moderately responsive, BW1, BW162, and BW 171are low responsive and BW 6 is non-responsive.
- Nine genotypes having common parentage (HD2967/HD2887//HD2946/HD2733) showed varied P response index. BW70, BW158 are responsive; BW174, BW155 are moderately responsive; BW124, BW167, BW151 are low responsive and BW139, BW2 are non-responsive.
- Five genotypes with parentage CSW3/HD2932+Yr10 showed varied P response index. BW 116 is responsive; BW 169 is moderately responsive; BW92, BW106 are low responsive; and BW136 is non-responsive.
