# Supplementary material for: Deciphering the change in root system architectural traits under limiting and non-limiting phosphorus in Indian bread wheat germplasm
Source: PLoS One. 2021 Oct 1;16(10):e0255840. doi: 10.1371/journal.pone.0255840 (PMC8486105; doi:10.1371/journal.pone.0255840)
Supplement: Attachment — Submitted filename: Reviewer #2_Comments.docx [file pone.0255840.s004.docx]

**Authors response to Reviewer #2 comments**

**General comment**

1. The work related to Root System Architecture (RSA) under Phosphorous limited conditions in wheat has not been studied comprehensively due to its difficulty in phenotyping. The manuscript reports identification of RSA traits and genotypes influencing P use efficiency under hydroponics conditions, which I feel a reliable strategy and could be used effectively for screening. These P efficient genotypes, as they are advanced and fixed lines, could serve as excellent donors in developing P efficient wheat genotypes.

However, the manuscript need improvement especially in discussion part.

**Authors**: The discussion part has been improved, as per the suggestions and new information is incorporated to make discussion part more readable.

I would suggest moderate revision for this manuscript.

**Authors**: The manuscript has been subjected to an additional step of proof reading and the modifications are made wherever needed to improve the overall impact of the manuscript.

**Introduction**

2. Introduction is well written. The importance of P in improving the productivity is well explained. However, authors should explain more about in RSA traits under P efficient and P limited conditions. If studies are limited in Wheat, other cereal crops may be explored like maize, rice etc. In rice, good number of papers are available on RSA traits influenced by P use efficiency (e.g. The role of root size versus root efficiency in phosphorus acquisition in rice. Mori et al 2013 Journal of Experimental Botany,2016, 67:1179–1189).

**Authors**: The Authors have realised the importance of this comment and included a paragraph explaining importance of RSA traits in improving productivity. In addition, the studies in rice and *Arabidospsis* have been discussed to have a comparative understanding on the root trait systems in wide range of crops.

**Material and Methods**

3. 182 advanced breeding lines comprising of diverse parentages is a good experimental material for this study. Statistical analysis is appropriate. Comprehensive phosphorus response index value is well calculated and is important identifying P responsive and non-responsive genotypes. Authors have not included released varieties in the experimental material Why?

**Authors**: We have used three popular, high yielding commercial varieties viz., HD2967, HD3226 and HDCSW18 and the comprehensive phosphorus response index values for these varieties is provided in the Table 5. These varieties have been bred under optimum P environment and released for the same. Therefore, they have low PUE. And hence, we have not considered them for the statistical analysis and the interpretations.

These commercial varieties were also used as the test material because of their change in root system architecture under P stress was not studied earlier to make them as check to compare with other genotypes. Unless their performance proved, we are unable to make use in comparison. We did not disclose commercial varieties data, which we need to further analyse to study the change in the PUE of commercial varieties released over period of time.

**Result**

4. The results are well presented. Subtitles should not be lengthy. The authors have written important findings in the subtitles which I feel quite good however, it will be confusing for the readers because of their lengthiness. Try to make it concise where it is possible or give name of the analysis as subtitle.

**Authors**: Subtitles have been concised for easy understanding.

5. In PCA, Figure 3B. the angle of eigen vectors between TRV, TRL, PRL, TRT, TRF and TSA is less than 90^o^, it indicates they are quite related to each other and we can take most important trait for the study leaving other less important traits. Which trait do you think is more important under limited P conditions and you advocate in the abstract for the readers?

**Authors**: Our results suggested that TRL, TSA, TRV and TRF were sufficient to explain the most of variation and these were proved to be ideal traits for phosphorus uptake efficiency screening at seedling stage. As most of these traits showed very close relatedness in terms of explaining the variation we, can choose TRL as the main trait as it is a combination of many component RSA traits determining the efficiency of genotypes.

**And the same information is incorporated in the main text under the discussion part.**

6. In title of figure 5 for better clarity, rather than writing RSA of contrasting genotypes, write better performing (BW 181 and BW 103) and poor performing (BW 9 and BE 139) genotypes grown under non-limiting (NLP) and limiting (LP) phosphorus conditions.

**Authors**: Appropriate changes have been made in the title of the Fig. 5 as per the suggestion.

7. In P efficient genotypes, viz., BW181, BW103, and BW66 a common parent noticed is HDCSW 18. This is a very high yielding wheat variety developed by IARI, New Delhi for conservation agriculture conditions. It would be great if authors could write more about this variety in relation to its P or N use efficiency in results as well as in discussion.

**Authors:** In our findings, HDCSW18 was identified as low P responsive because it is having high TRL, PRL, TSA, TRF and TRT values in both P regimes. That means this variety might be having an inherent capacity to show constant response to changing P regimes for RSA traits. Under CA environments the nutrient use efficiency is low as compared to normal tillage conditions. Because the applied fertiliser is retained for longer time on the soil surface and the plants should have the inherent capacity to absorb the nutrients from the soil rather than totally dependent on the external supply of fertiliser. HDCSW18 has been bred under Conservation Agriculture (CA) environments and therefore believed to be having better absorption capacity for the nutrients due to its strong RSA traits.

**This information is incorporated in the discussion part of the manuscript.**

8. Similarly, in the parentage of P non-efficient wheat genotypes like BW 9, BW 139, BW 3, two mega wheat varieties HD 2967 and HD 2733 are involved. If authors would come out with a reason why recombinants of these best genotypes adapted to farmers field showed poor P efficiency?

**Authors**: HD2967 and HD2733 are bred and released under optimum P environments. In our analsysis HD 2967 showed low P response for P use efficiency. We have not tested the same for HD 2733, but, we assume that, it must be lacking inherent capacity to respond same under varying P regimes.

9. If authors would have involved released varieties (popular varieties like HDCSW 18, HD 2967, HD 3086, HD 2733 etc.) in this study, it would have been good information.

**Authors**: We do have generated data on few of the released varieties like HD2967, HD3226 and HDCSW18 and the same has been included in the Table No. 5.

**Discussion**

10. Discussion is quite lengthy and need to be revised. Results are repeated in the discussion which is not required. Authors can cite references from other cereal crops pertaining to RSA under different nutrient stresses.

**Authors:** The discussion part has been improved, through incorporating information on other cereal crops in terms of their studies on RSA traits. The text in the discussion has be concised to increase the readability and to keep the information crisp and to the point.

11. In the discussion, author may give statements on genetics of P use efficiency in studied genotypes based on their parentages

**example:**

Six genotypes shared common parentage (CSW2/ HD2932+Yr15); however, they differ for their P response index. BW143 is highly responsive, BW69 is moderately responsive, BW1, BW162, and BW 171are low responsive and BW 6 is non-responsive.

**Authors:** Its very interesting to note that, the six genotypes mentioned here are sharing a common parent CSW2 and HD2932+Yr15 and hence, this becomes more obvious to accept the fact that, both the parents are contrasting for P use efficiency. And it shows the P use efficiency is polygenic in nature. These lines may be suggestive to be used as parents in developing mapping populations for characterising RSA traits

**This information has been incorporated in the main text under discussion part.**

Nine genotypes having common parentage (HD2967/HD2887//HD2946/HD2733) showed varied P response index. BW70, BW158 are responsive; BW174, BW155 are moderately responsive; BW124, BW167, BW151 are low responsive and BW139, BW2 are non-responsive.

**Authors:** As discussed above, the nine genotypes mentioned here are sharing a common parent and hence, again this becomes more obvious to accept the fact that, both the parents are contrasting for P use efficiency. And it shows the P use efficiency is polygenic in nature.

**This information has been incorporated in the main text under discussion part.**

Five genotypes with parentage CSW3/HD2932+Yr10 showed varied P response index. BW 116 is responsive; BW 169 is moderately responsive; BW92, BW106 are low responsive; and BW136 is non-responsive.

**Authors:** Again these genotypes also share a common parents and transgressive segregants may be the reason for varied responses
